# Iron deficiency associates with deterioration in several symptoms independently from hemoglobin level among chronic hemodialysis patients

**DOI:** 10.1371/journal.pone.0201662

**Published:** 2018-08-02

**Authors:** Shuta Motonishi, Kentaro Tanaka, Takashi Ozawa

**Affiliations:** 1 Kodaira Kitaguchi Clinic, Tokyo, Japan; 2 Higashikurume Ekimae Clinic, Tokyo, Japan; Pennsylvania State University College of Medicine, UNITED STATES

## Abstract

**Background:**

While iron deficiency (ID) is a frequent cause of anemia in hemodialysis patients, the clinical impact of ID without anemic level of hemoglobin remains unclear. As such, this study was designed to clarify the manifestations of ID itself in subjects on hemodialysis.

**Methods:**

Maintenance hemodialysis patients achieving target hemoglobin levels (≥ 10.0g/dL) under treatment in our clinic were stratified for comparison from three perspectives: ID (transferrin saturation [TSAT] < 20% or ferritin < 100ng/mL) vs non-ID, level of TSAT (< or ≥ 20%), and level of serum ferritin concentration (< or ≥ 100ng/mL). The severity of frequent symptoms was determined by a self-rating symptom score questionnaire, and the rate of those with severe manifestations was calculated for each symptom. Significant difference was examined between groups; univariate and adjusted multivariate odds ratios and 95% confidence intervals were obtained by logistic regression.

**Results:**

Among 154 subjects selected for analysis, the ratio of severe arthralgia and fatigue was significantly higher in the ID group (n = 94) compared to the non-ID group (n = 60), in both univariate and adjusted multivariate analyses. Moreover, in multivariate analysis, low TSAT was significantly associated with exacerbation of pain during vascular access puncture and intradialytic leg cramps, while low serum ferritin concentration was related to significant increase in severe arthralgia, fatigue, intradialytic headache and leg cramps.

**Conclusions:**

ID was identified as a risk factor regarding severity of several symptoms even without low hemoglobin level among chronic hemodialysis patients, and supplementation of iron was considered efficacious for improving critical symptoms affecting those undergoing maintenance dialysis.

## Introduction

Recent studies have revealed an association between iron deficiency (ID) and poor prognosis in chronic heart failure (CHF) regardless of anemic status, and that left ventricular performance could be improved by iron supplementation [1–3]. Given such findings, intravenous iron administration is now recommended for patients with CHF and ID according to the guideline of the European Society of Cardiology [4]. ID has also been associated with restless leg syndrome, mental disorders, fatigue and exercise intolerance, irrespective of hemoglobin (Hb) level [5–8].

ID is a common cause of erythropoiesis stimulating agent (ESA)-resistant anemia in patients with chronic kidney disease (CKD) including those undergoing chronic hemodialysis (HD). ID in the CKD patient is caused by a variety of factors including inadequate intake of iron due to appetite loss or dietary restrictions, frequent blood sampling for laboratory testing, chronic iron loss through intestinal hemorrhage induced by uremic platelet dysfunction, and frequent administration of anticoagulants for vascular complications [9,10]. Moreover, CKD patients often have elevated hepcidin levels that interfere with iron absorption and transfer by inhibiting ferroportin function [11,12]. In patients on HD, iron loss during HD also contributes to ID. On the other hand, a substantial number of HD patients achieving target Hb levels often develop ID. Despite this situation, the clinical impact of ID itself among HD patients has not been described to date, and the benefit of iron supplementation in HD patients with ID without reduction of Hb value has yet to be verified.

As such, chronic HD patients without anemic levels of Hb were examined to investigate the effects of non-anemic ID in this study. Patients were first classified into ID and non-ID (iron sufficient) groups, then in terms of transferrin saturation (TSAT) and serum ferritin levels. The groups were compared to clarify the relationship between ID and the severity of several general symptoms using a symptom score questionnaire constructed by Masakane [13], which is frequently used to assess the physical status of dialysis patients in Japan. The impact of low TSAT or low ferritin on symptom severity was similarly investigated. Odds ratios (ORs) and 95% confidence intervals (CIs) were calculated, and significant difference was confirmed using Fisher’s exact test. Furthermore, adjusted multivariate analysis was conducted by logistic regression adjusting for patient profile, dose of agents, and biochemical data. Adjusted multivariate ORs and 95% CIs were determined to confirm the independent impact of ID.

## Materials and methods

### Subjects

Prior to data collection for this study, all subjects were being treated according to guidelines advocated by The Japanese Society for Dialysis Therapy. Target iron parameters for this study were defined as ≥ 20% TSAT and ≥ 100ng/mL ferritin.

**Fig 1:**
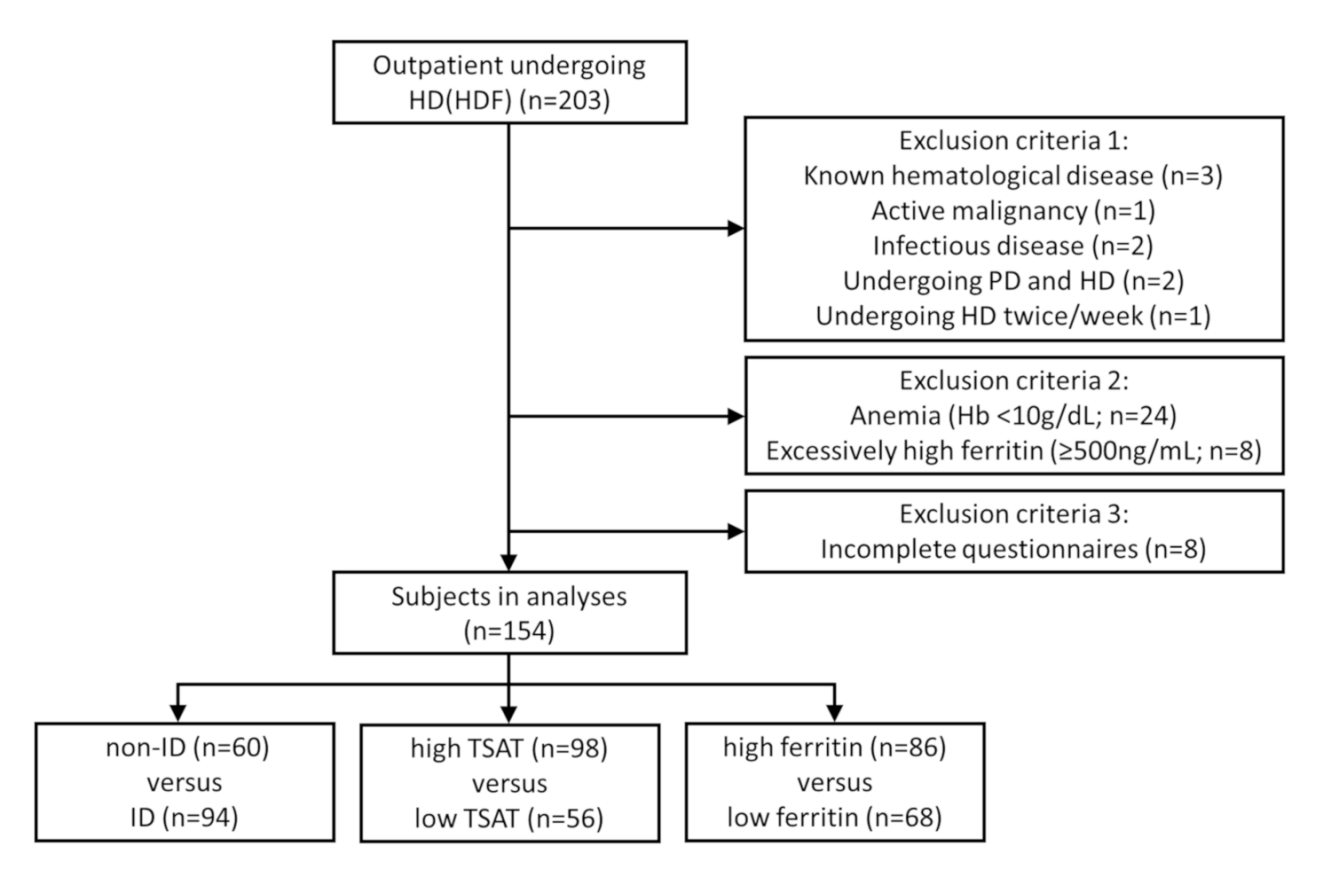
Flowchart of subject selection in this study. A total of 203 outpatients undergoing HD or HDF were examined, and 154 patients were analyzed after exclusion of subjects according to criteria as shown. HD: hemodialysis, HDF: hemodiafiltration, PD: peritoneal dialysis, Hb: hemoglobin, ID: iron deficiency, TSAT: transferrin saturation.

All out-patients undergoing HD or hemodiafiltration (HDF) three times/week at our institute were considered for this study (n = 203), from which subjects with known hematological disorders, active malignancies, and severe chronic infectious disease were excluded. We defined the “low Hb” (anemic) level was defined as that lower than 10.0g/dL, the condition requiring treatment for anemia in chronic HD patients, and 24 such patients were considered ineligible. A cutoff level of 500ng/mL ferritin was used to identify patients with excessively high serum ferritin concentrations (n = 8), who were also excluded assuming the presence of some physiology inducing impaired availability of iron. Furthermore, to equalize the effect of dialysis method among subjects, patients being treated by both hemodialysis and peritoneal dialysis or undergoing HD twice a week were also excluded from this study, as were those returning incomplete questionnaires (Fig 1). A total of 154 patients were thus included in the analysis. All were adults giving written consent regarding participation in the study.

### Definition of iron deficiency and classification of subjects

According to the latest 2016 guideline from The Japanese Society for Dialysis Therapy [14,15], iron supplementation is indicated when TSAT is < 20% and/or ferritin level is < 100ng/mL. In this study, we defined ID as the condition corresponding to either TSAT < 20%, serum ferritin < 100ng/mL, or both. The subjects were classified and analyzed from three perspectives regarding ID status as follows: (1) ID (TSAT < 20% or ferritin < 100ng/mL) vs non-ID (TSAT ≥ 20% and ferritin ≥ 100ng/mL); (2) low TSAT (< 20%) vs high TSAT (≥ 20%); and (3) low ferritin (< 100ng/mL) vs high ferritin (≥ 100ng/mL). Patient group characteristics and analysis results are presented per these three perspectives.

### Data collection

For measurement of biological and hematological data, blood sampling was performed before the first HD session of the week (Monday or Tuesday), two days from the previous HD. Dose of administered ESAs, drugs for iron supplementation (both oral and intravenous), duration of HD, and other patient profiles were obtained from medical records. All data sampling was conducted in April 2016, during which symptom score questionnaires were also completed by the patients and retrieved.

### Assessment of symptoms

**Fig 2:**
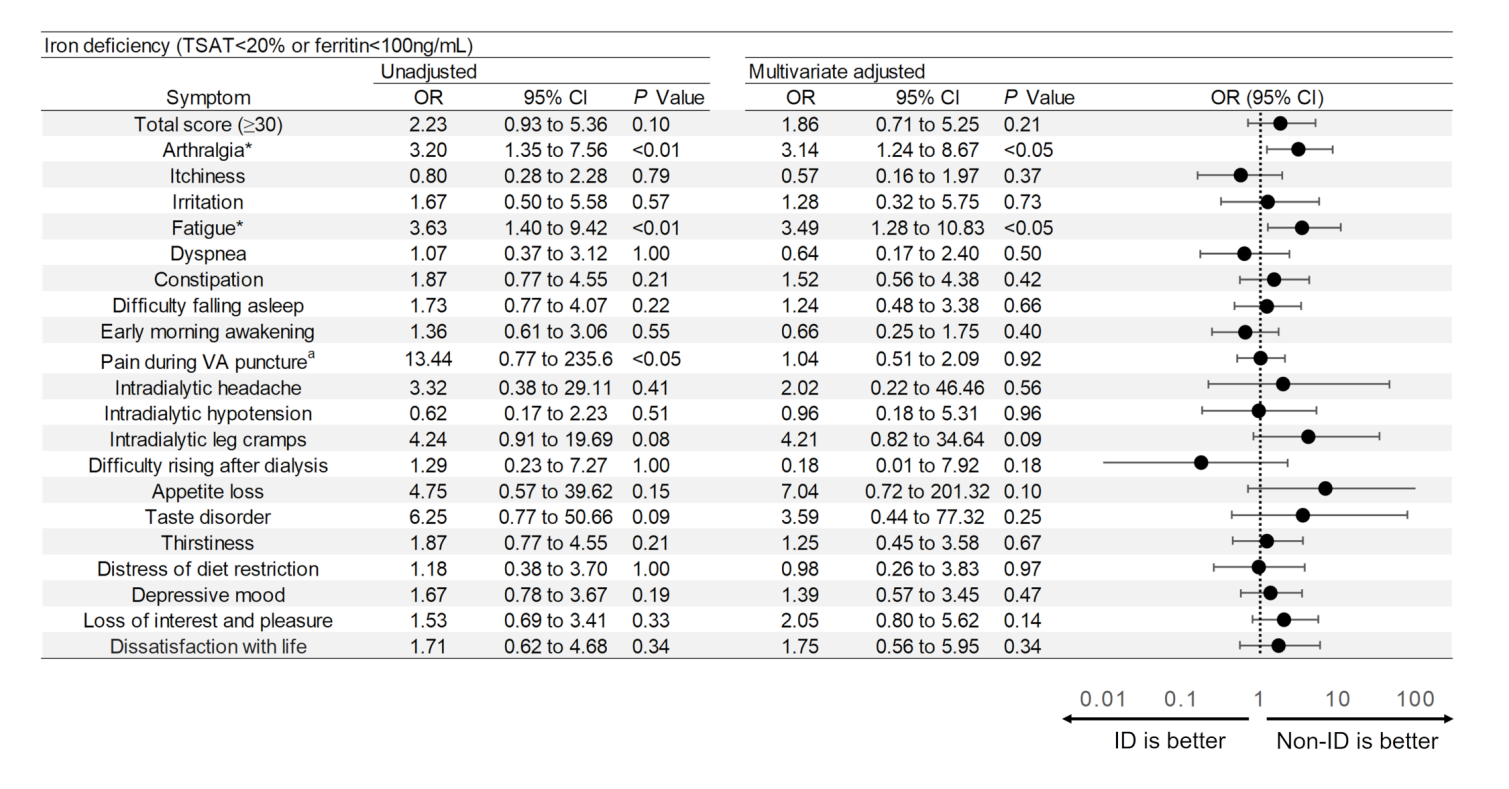
Odds ratios and forest plot for severity of symptoms in ID patients (vs non-ID). Multivariate ORs and 95% CIs were calculated using logistic regression analysis with adjustment for age, gender, duration of dialysis, presence or absence of diabetes, dose of iron, dose of erythropoiesis-stimulating agents, serum albumin level and serum β2 microglobulin level. a: Iron sufficient patients with severe symptoms were not found, and odds ratios were calculated by adding 0.5 to each value. *: P value <0.05 for multivariate adjusted analysis. TSAT: transferrin saturation, VA: vascular access.

**Fig 3:**
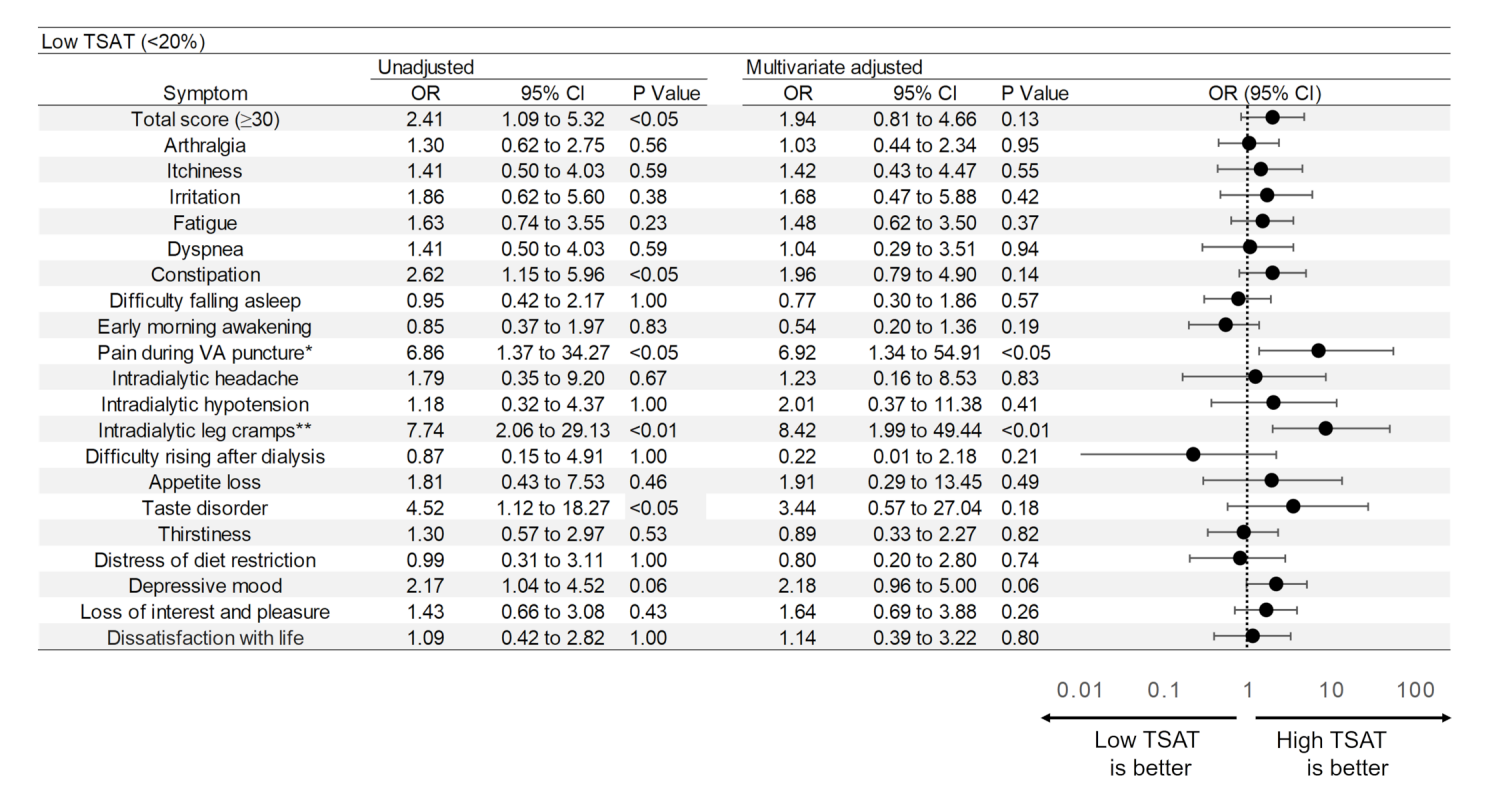
Odds ratios and forest plot for severity of symptoms in patients with low TSAT (vs high TSAT). Multivariate ORs and 95% CIs were calculated using logistic regression analysis with adjustment for age, gender, duration of dialysis, presence or absence of diabetes, dose of iron, dose of erythropoiesis-stimulating agents, serum albumin level, serum β2 microglobulin level and serum ferritin level. *: P value <0.05, **: P value <0.01 for multivariate analysis. TSAT: transferrin saturation, VA: vascular access.

**Fig 4:**
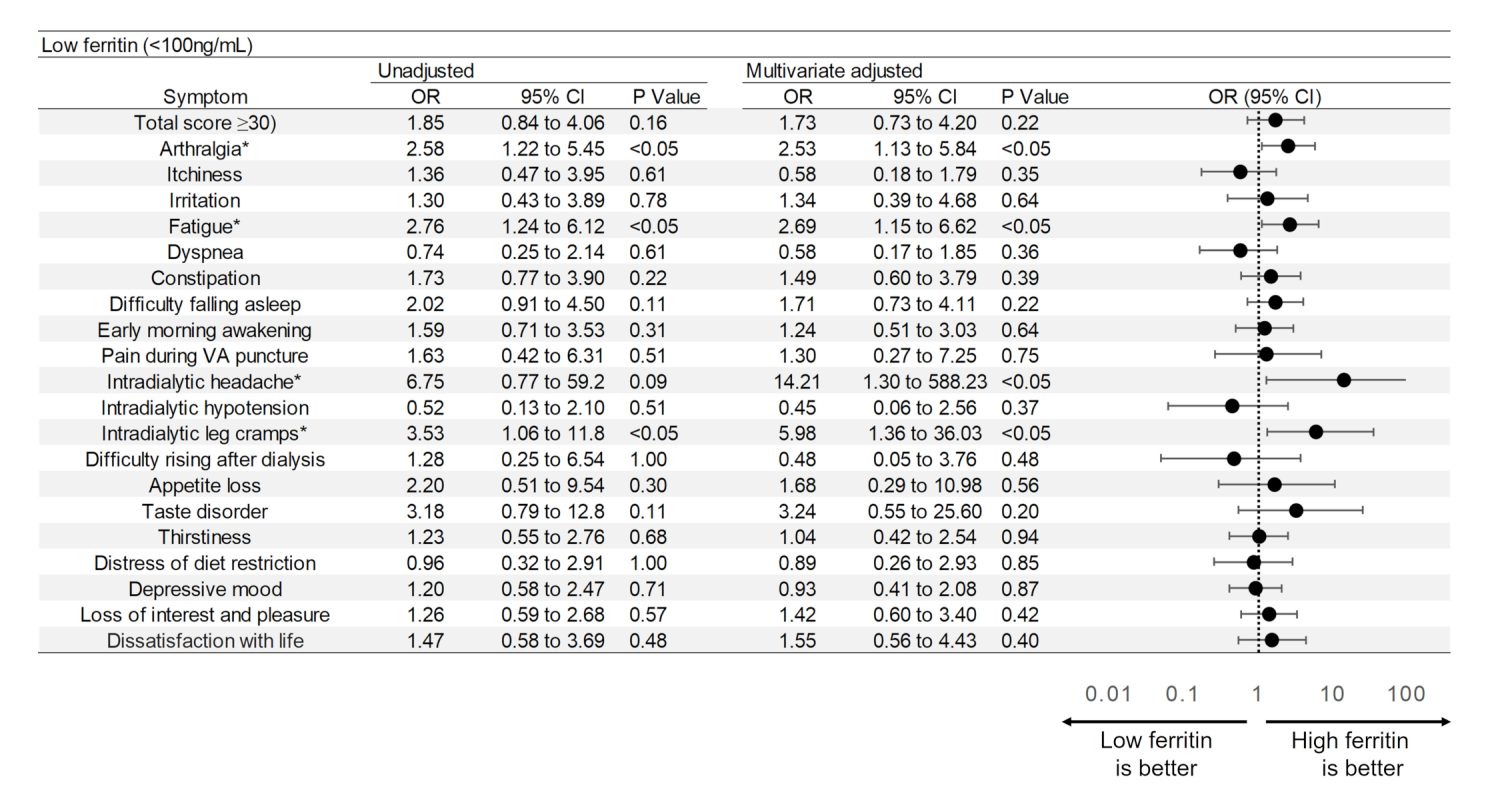
Odds ratios and forest plot for severity of symptoms in patients with low ferritin (vs high ferritin). Multivariate ORs and 95% CIs were calculated using logistic regression analysis with adjustment for age, gender, duration of dialysis, presence or absence of diabetes, dose of iron, dose of erythropoiesis-stimulating agents, serum albumin level, serum β2 microglobulin level and serum TSAT level. *: P value <0.05 for multivariate analysis. TSAT: transferrin saturation, VA: vascular access.

Physical and mental conditions of the patients were assessed using a symptom score questionnaire (S1 Fig.) constructed by Masakane [13], which is frequently used to investigate physical and mental status of dialysis patients in Japan. His therapeutic concept—the Patient-oriented Dialysis (POD) system—defines good dialysis as that free of uncomfortable dialysis-related symptoms, in which consideration for patient views and wishes regarding dialysis and daily life is a paramount concern. Concurring with this concept, the self-rating score questionnaire developed for the POD system was adopted for symptom assessment in our study. The questionnaire (S1 Fig) consists of 20 items addressing general symptoms in HD patients (as listed in Figs 2–4) scored on a 5-step scale from 0 (mildest) to 4 (most severe). For statistical analysis, scores of 0–2 were regarded as mild symptoms and scores 3–4 as severe. Two of the 20 items (depressive mood, loss of interest and pleasure) calling for yes/no responses representing presence/absence of symptoms were scored as 4 = present or 0 = absent. Total scores (sum of all scores) were calculated as overall indication of the patient’s condition.

### Statistical analysis

All numerical data are reported as means ± standard deviation (SD) excepting ratios among groups. For each symptom, the number of patients with either mild or severe symptoms were counted and compared between groups (iron deficient vs sufficient, low vs high TSAT, or low vs high ferritin) using Fisher’s exact test, ORs and 95% CIs. Adjusted multivariate data was obtained by logistic regression with adjustment for patient profiles and clinical conditions including gender, age, duration of hemodialysis, percentage of patients with diabetes, dose of ESA, dose of iron, serum albumin and serum β_2_ microglobulin levels. To compare between low and high TSAT groups, serum ferritin level was also adjusted as a confounding factor. To compare between low and high ferritin groups, TSAT was conversely adjusted for analysis. The strength of linear association between two values was measured using Spearman’s correlation coefficient. Differences with a P value of < 0.05 were considered significant. GraphPad Prism software (version 5.04 for Windows, GraphPad Software, San Diego, CA, USA) and JMP software (version 9.0.2, SAS Institute, Cary, NC, USA) were used.

### Study approval

This study was conducted in accordance with the Declaration of Helsinki, with approval by the Clinical Research Ethics Committee of Medical Toyou (Kanagawa Prefecture, Japan), on patients giving signed informed consent.

## Results

### Selection of subjects and baseline analysis

Following exclusion of patients as described under Materials and Methods and Fig 1, 154 subjects were included for analysis in this study. Mean age was 65.0 ± 12.8 years old, including 41 (26.6%) female and 113 (73.4%) male patients. Mean duration of HD was 7.4 ± 6.8 years. The number of patients with diabetes mellitus was 56 (36.4%). Among all patients, mean TSAT and ferritin level was 24.0 ± 10.0% and 156.3 ± 128.1ng/mL, respectively. The subjects were classified and compared regarding iron deficiency from three perspectives depending on TSAT and serum ferritin levels.

### Comparison between ID and non-ID groups

The baseline characteristics of non-ID and ID patient groups are shown in Table 1. Except for iron parameters, most data were comparable between groups, suggesting no association between these indices and the following differences in severity: doses of erythropoiesis-stimulating agents (ESAs) were significantly higher in the ID group—consistent with previously reported findings [16] that ID reduced responsiveness to ESAs resulting in need for dose increments to maintain target Hb levels—and duration of HD was longer in the non-ID group.

**Table 1:** Baseline characteristics of non-ID and ID groups.

	Non-ID (N = 60)	ID (N = 94)	*P* Value
	(TSAT≥20 and Fer≥100)	(TSAT<20 or Fer<100)	
Gender (% Female)	23.0	28.7	0.46
Age (years)	66.1±13.3	64.1±12.8	0.51
Hemodialysis duration (years)	8.3±7.6	6.0±5.1	0.037
Diabetes (%)	36.7	36.2	1.00
Dose of ESA(U/week) ^a^	2138±1504	4005±2960	<0.001
Dose of iron (mg/week)	9.4±20.0	3.9±15.8	0.055
Hemoglobin (g/dL)	11.3±1.0	11.3±1.0	0.59
Hematocrit (%)	35.3±3.3	35.7±3.3	0.59
Fe (μg/dL)	65.3±25.2	53.6±21.0	<0.001
TIBC (μg/dL)	221.5±47.5	259.7±29.2	<0.001
TSAT (%)	29.7±9.7	20.8±9.9	<0.001
Ferritin (ng/mL)	261.3±107.3	98.6±110.6	<0.001
Albumin (g/dL)	3.66±0.31	3.74±0.26	0.085
Creatinine (mg/dL)	10.8±2.5	10.5±2.7	0.59
BUN (mg/dL)	62.8±14.7	61.7±12.7	0.63
K (mEq/L)	4.8±0.6	4.9±0.6	0.39
cCa (mg/dL) ^b^	9.1±0.7	9.0±0.6	0.61
P (mg/dL)	5.1±1.2	5.1±1.0	0.98
β2MG (mg/L)	27.4±6.5	26.6±6.3	0.46

Values are means ± SD, or ratios regarding gender and diabetes. All data are concentrations in serum before dialysis, 2 days from the previous session.

^a^: Unit of epoetin alfa equivalent.

^b^: Corrected calcium was calculated according to the equation: corrected calcium = measured calcium + (4.0—albumin (g/dL)) when albumin was lower than 4.0g/dL.

TSAT: transferrin saturation, Fer: ferritin, ESA: erythropoiesis-stimulating agent, Fe: iron, TIBC: total iron-binding capacity, BUN: blood urea nitrogen, K: potassium, cCa: corrected calcium, P: phosphorus, β2MG: β_2_ microglobulin.

ORs and 95% CIs for all scored symptoms are presented in Fig 2. Among the 20 symptoms, the rates of patients with severe arthralgia (OR 3.20, 95%CI 1.35–7.56) and fatigue (OR 3.63, 95%CI 1.40–9.42) were significantly higher in the ID group than the non-ID group by Fisher’s exact test. The ratio of patients with high total score (OR 2.23, 95%CI 0.93–5.36, P = 0.10), those with severe intradialytic leg cramps (OR 4.24, 95%CI 0.91–19.69, P = 0.08) and those with severe taste disorder (OR 6.25, 95%CI 0.77–50.66, p = 0.09) also tended to be higher in the ID group. Adjusted multivariate data calculated by logistic regression also yielded a significantly higher ratio of patients with severe symptoms in the ID group regarding arthralgia (OR 3.14, 95%CI 1.24–8.67) and fatigue (OR 3.49, 95%CI 1.28–10.83), as well as a tendency for severe appetite loss (OR 7.04, 95%CI 0.72–201.32, P = 0.10).

### Comparison between low and high TSAT groups

Baseline characteristics were similar except for iron parameters such as serum iron, total iron-binding capacity (TIBC), TSAT, and ferritin, and the dose of ESAs being administered, which was significantly lower in the high TSAT group (Table 2).

**Table 2:** Baseline characteristics of high TSAT and low TSAT groups.

	TSAT≥20%	TSAT<20%	*P* Value
	(N = 98)	(N = 56)	
Gender (% Female)	25.3	30.4	0.46
Age (years)	64.3±12.6	66.2±13.1	0.36
Hemodialysis duration (years)	6.76±6.2	8.1±7.4	0.23
Diabetes (%)	33.7	41.2	0.39
Dose of ESA(U/week) ^a^	2776±2022	4228±3496	0.0013
Dose of iron (mg/week)	7.8±20.5	2.9±10.4	0.094
Hemoglobin (g/dL)	11.3±1.0	11.2±1.0	0.41
Hematocrit (%)	35.4±3.3	35.7±3.2	0.57
Fe (μg/dL)	69.1±21.5	38.0±11.2	<0.001
TIBC (μg/dL)	237.9±40.7	257.7±50.3	<0.001
TSAT (%)	29.2±8.6	14.9±3.7	<0.001
Ferritin (ng/mL)	180.0±135.3	114.8±103.0	0.0021
Albumin (g/dL)	3.71±0.30	3.70±0.26	0.94
Creatinine (mg/dL)	10.8±2.6	10.4±2.6	0.38
BUN (mg/dL)	61.9±13.0	62.6±14.4	0.78
K (mEq/L)	4.83±0.6	4.86±0.70	0.72
cCa (mg/dL) ^b^	9.1±0.6	9.1±0.6	0.97
P (mg/dL)	5.1±1.1	5.0±0.9	0.88
β2MG (mg/L)	26.6±6.2	27.4±6.6	0.45

Values are means ± SD, or ratios regarding gender and diabetes. All data are concentrations in serum before dialysis, 2 days from the previous session.

^a^: Unit of epoetin alfa equivalent.

^b^: Corrected calcium was calculated according to the equation: corrected calcium = measured calcium + (4.0—albumin (g/dL)) when albumin was lower than 4.0g/dL.

TS0041T: transferrin saturation, ESA: erythropoiesis-stimulating agent, Fe: iron, TIBC: total iron-binding capacity, BUN: blood urea nitrogen, K: potassium, cCa: corrected calcium, P: phosphorus, β2MG: β_2_ microglobulin.

**Fig 5:**
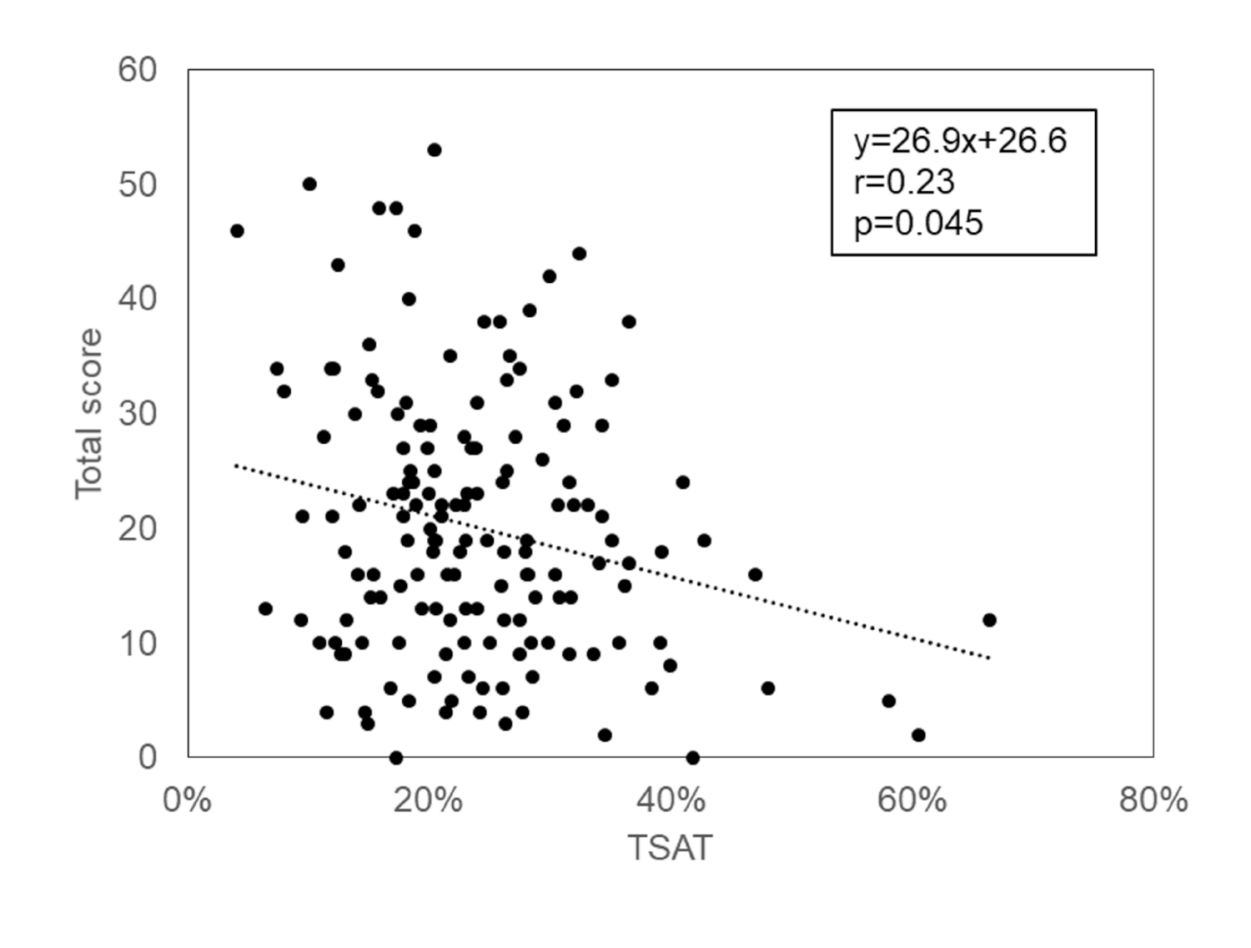
Relationship between TSAT and total score. The strength of linear association between TSAT and total symptom questionnaire score was analyzed by Spearman’s correlation coefficient. TSAT: transferrin saturation.

The results of unadjusted and adjusted multivariate analyses are shown in Fig 3. Significant differences were detected in high total score (OR 2.41, 95%CI 1.09–5.32), constipation (OR 2.62, 95%CI 1.15–5.96), pain during vascular access (VA) puncture (OR 6.86, 95%CI 1.37–34.27), intradialytic leg cramps (OR 7.74, 95%CI 2.06–29.13) and taste disorder (OR 4.52, 95%CI 1.12–18.27) in univariate analysis. In the adjusted multivariate analysis with adjustment for ferritin level, the ratio of patients with severe symptoms was significantly higher in the low TSAT group regarding pain during VA puncture (OR 6.92, 95%CI 1.34–54.91) and intradialytic leg cramps (OR 8.42, 95%CI 1.99–49.44). Moreover, the ratio of subjects with severe depressed mood tended to be higher in the low TSAT group both in unadjusted (OR 2.17, 95%CI 1.04–4.52, p = 0.06) and adjusted multivariate analysis (OR 2.18, 95%CI 0.96–5.00, p = 0.06). In univariate analysis, we demonstrated that low TSAT was associated with high total score. In agreement with this result, significant correlation was found between TSAT and total score of the symptom score questionnaire using Spearman’s test (Fig 5).

### Comparison between low and high ferritin groups

Baseline characteristics are shown in Table 3. Among the profiles, difference was noted in serum albumin concentration, dose of ESA and dose of iron between the two groups in addition to the iron parameters—all factors adjusted for in multivariate analysis.

**Table 3:** Baseline characteristics of high ferritin and low ferritin groups.

	Ferritin≥100ng/mL	Ferritin<100ng/mL	*P* Value
	(N = 86)	(N = 68)	
Gender (% Female)	24.4	29.4	0.58
Age (years)	64.6±13.9	65.4±11.4	0.69
Hemodialysis duration (years)	6.7±6.3	8.2±7.4	0.17
Diabetes (%)	36.1	36.8	1.00
Dose of ESA(U/week) ^a^	2855±2499	3871±2927	0.021
Dose of iron (mg/week)	8.0±18.3	3.5±16.6	0.012
Hemoglobin (g/dL)	11.3±1.0	11.3±1.0	0.75
Hematocrit (%)	35.5±3.3	35.5±3.3	0.94
Fe (μg/dL)	56.4±23.0	59.6±24.8	0.41
TIBC (μg/dL)	225.0±33.5	270.5±45.6	<0.001
TSAT (%)	25.3±10.1	22.3±8.9	0.063
Ferritin (ng/mL)	242.2±109.3	47.6±28.8	<0.001
Albumin (g/dL)	3.65±0.30	3.77±0.25	0.012
Creatinine (mg/dL)	10.6±2.5	10.6±2.7	0.98
BUN (mg/dL)	62.9±15.4	61.1±10.7	0.41
K (mEq/L)	4.8±0.6	4.8±0.6	0.88
cCa (mg/dL) ^b^	9.1±0.6	9.0±0.6	0.90
P (mg/dL)	5.1±1.1	5.1±1.0	0.91
β2MG (mg/L)	27.1±6.0	26.7±6.8	0.66

Values are means ± SD, or ratios regarding gender and diabetes. All data are concentrations in serum before dialysis, 2 days from the previous session.

^a^: Unit of epoetin alfa equivalent.

^b^: Corrected calcium was calculated according to the equation: corrected calcium = measured calcium + (4.0—albumin (g/dL)) when albumin was lower than 4.0g/dL.

ESA: erythropoiesis-stimulating agent, Fe: iron, TIBC: total iron-binding capacity, TSAT: transferrin saturation, BUN: blood urea nitrogen, K: potassium, cCa: corrected calcium, P: phosphorus, β2MG: β_2_ microglobulin.

As shown in Fig 4, the low ferritin group was associated with higher severity of arthralgia (OR 2.58, 95%CI 1.22–5.45), fatigue (OR 2.76 95%CI 1.24–6.12) and intradialytic leg cramps (OR 3.53, 95%CI 1.06–11.8) in unadjusted analysis. In the adjusted multivariate analysis, patients with low ferritin indicated higher degrees of severity for arthralgia (OR 2.53, 95%CI 1.13–5.84), fatigue (OR 2.69, 95%CI 1.15–6.62), intradialytic headache (OR 14.21, 95%CI 1.30–588.23) and intradialytic leg cramps (OR 5.98, 95%CI 1.36–36.03).

## Discussion

While ID is one of the most frequent complications inducing ESA-resistant anemia among patients with end-stage renal disease (ESRD), a substantial proportion of patients exhibit ID with target Hb levels. Nevertheless, while various guidelines for anemia recommend iron administration upon determination of ID, there have been no reports clarifying the necessity or significance of treating non-anemic ID by iron administration to date.

This is the first study to examine and reveal the relationship between ID without low Hb and critical symptoms in HD patients. The novelty of this study was that all subjects were HD patients without reduced Hb values, enabling evaluation of the effects of ID independently of the anemic condition. Using this design, we found that ID was significantly associated with higher severity of arthralgia and fatigue, in both univariate (unadjusted) and adjusted multivariate analyses. In addition, we demonstrated that low TSAT was linked to severity of pain during vascular access (VA) puncture and intradialytic leg cramps, and that low ferritin level was associated with greater severity of arthralgia, fatigue, intradialytic headache and leg cramps. Importantly, these associations were confirmed by adjusted multivariate analysis including adjustments for dose of ESAs—a crucial confounding factor leading to difficulties in interpretation of results in many studies—as well as β_2_-microglobulin and albumin levels, essential for eliminating the effects of inflammation and malnutrition. Taken together, we believe our findings can provide novel insights for refining therapeutic strategy regarding ID in patients undergoing HD.

We have found no similar reports in patients with CKD including ESRD, whereas the association between ID and exacerbation of symptoms have been reported in some other distinct contexts. Comín-Colet et al. have indicated ID as a critical determinant of health-related quality of life in CHF patients [3]. Although their ID group included more anemic subjects than the non-ID group, they showed that ID was significantly associated with higher rates of patients with impaired physical activity, appetite loss, fatigue and shortness of breath, which were similar to our results. Brownlie et al. demonstrated that non-anemic ID was linked to impaired adaptation in endurance capacity after aerobic training, and that iron supplementation significantly enhanced adaptation in both maximal work capacity and endurance [5,17,18]. Similar results have been shown in earlier studies on animals [19,20] and humans [21], while Sawada and colleagues have gone on to associate ID with not only fatigue, but with anger and tension in women of childbearing age [6]. Our findings were consistent with these many studies regarding higher severity of fatigue and appetite loss.

Although the major proportion of iron is taken up by erythroblasts and reticulocytes for Hb synthesis [22,23], iron is indispensable for the maintenance of cellular energy, and metabolism of extra-hematopoietic tissue. Iron plays a crucial role in oxygen transport (Hb component), oxygen storage (myoglobin component), cardiac and skeletal muscle metabolism (oxidative enzyme and respiratory chain protein components) and mitochondrial function [2,24–27]. Therefore, it is natural that iron insufficiency should cause impairment in exercise capacity and endurance resulting in increased fatigue, even when it does not reach levels of apparent anemia. Recent papers have demonstrated that ID is associated with severity of cardiac function and poor prognosis in CHF patients regardless of Hb value [2,3,28,29]. Because CHF is a highly prevalent complication in HD patients [30,31], ID-involved deterioration of CHF may also be contributing to exacerbation of fatigue and lowering of exercise capacity among patients undergoing HD.

With regards to mental disorders, our low TSAT group exhibited a higher tendency for severe depressed mood. Similar results have been reported in subjects with heart failure (HF) by Jankowska et al. [2]. Interestingly, they reported association of ID with more severe depressive symptoms independently from Hb level, HF severity, neurohormonal activation and inflammation, suggesting that ID itself may be capable of inducing the psychological disorder. As one of the mechanisms behind such findings, previous studies have reported on the important role played by iron in neurotransmitter synthesis, uptake and degradation. Iron is also known for its critical role in mitochondrial function, richly distributed within the metabolically active neurons in brain tissue [2,32].

Regarding neurological alteration, prior studies have shown that ID may cause defects in dopaminergic interaction with the opiate system and cholinergic neurotransmission [33], which might explain our findings regarding exacerbation of arthralgia, headache, and pain during Vascular access puncture. In fact, previous reports from animal experiments suggest that ID can increase sensitivity to pain [32,34,35]. This association has been attributed to hyperactivity of neurons in the dorsal horn of the spinal cord, and/or alteration of dopamine neurotransmission in the central nervous system. Kallianpur et al. [36] have reported that genetic variation in iron-regulation was associated with neuropathic pain severity in HIV-infected patients. The increase in severe leg cramps reported in our study is thought to be due not only to heightened sensitivity to pain, but also to energy metabolism disorders affecting the skeletal muscle of the legs.

The use of iron is increasing in accordance with renal anemia guidelines in the Western countries, largely due to the decreased ESA usage without bundled payment methods given worse outcomes following excessive dosing of ESAs [16,37,38]. In contrast, Japanese guidelines have been very restrictive in the prescription of iron. Until recently, iron administration was permitted in HD patients only when TSAT < 20% AND ferritin < 100ng/mL. This was partly due to concern over intravenous iron preparations increasing oxidative stress and iron accumulation in the liver, leukocytes and cardiovascular system. Considering that the range of serum ferritin level is much lower in Japanese HD patients than patients in the West [39], the benefits of iron supplementation was in dire need of review. The newest Japanese guideline published in 2016 now allows for iron supplementation when TSAT < 20% OR ferritin < 100ng/mL, with the proviso that patients must be free of pathophysiology causing impaired availability of iron [14,15]. Our findings from the present study support this modification.

This study is subject to a number of limitations. First, our results are a post-hoc, cross-sectional analysis of data from a single center, making it unclear whether the findings are applicable to other facilities, countries and ethnicities. On the other hand, adequate demonstration of causality is generally difficult, for instance, stronger fatigue could well be regarded as a causative factor inducing appetite loss leading to inadequate iron intake. However, our results—confirmed by multivariate analysis adjusting for serum albumin to eliminate malnutrition as a confounding factor—were able to indicate otherwise. The only difference found in comparing albumin levels at baseline was in the comparison between high and low ferritin groups, in which albumin concentration was lower in the high ferritin group, suggesting insignificance of the deleterious effects of malnutrition. Second, our symptoms data was entirely subjective being collected using a self-reporting symptom score questionnaire, which does not allow for objective evaluation even with care to avoid unnecessary bias by eliminating subjects returning incomplete questionnaires from the analysis. Finally, our study did not include investigation into the effects of iron supplementation. Nevertheless, our findings did demonstrate that ID was significantly related to a number of critical symptoms, which strongly supports iron supplementation to maintain optimal levels of health-related quality of life, even in patients with non-anemic ID. Future study is needed to examine the relationship between ID and symptoms using more accurate methodology for assessment of the effects of iron administration.

In conclusion, we revealed that deficiency in iron is significantly associated with severity of general symptoms independently of Hb level, suggesting that ID is a critical risk factor for deterioration of physical and mental conditions in maintenance HD patients. We believe our findings are of value in contributing to the understanding of ID and refinement of therapeutic strategy regarding iron supplementation in patients undergoing HD.

## Supporting information

S1 Fig Self-rating symptom score questionnaire. (PDF)

S1 Table Data sheet on all analyzed subjects. (XLSX)
