# Correction: Iron deficiency associates with deterioration in several symptoms independently from hemoglobin level among chronic hemodialysis patients

**DOI:** 10.1371/journal.pone.0204789

**Published:** 2018-09-24

**Authors:** Shuta Motonishi, Kentaro Tanaka, Takashi Ozawa

Fig 5 is incorrect. The authors have provided a corrected version here.

**Fig 5:**
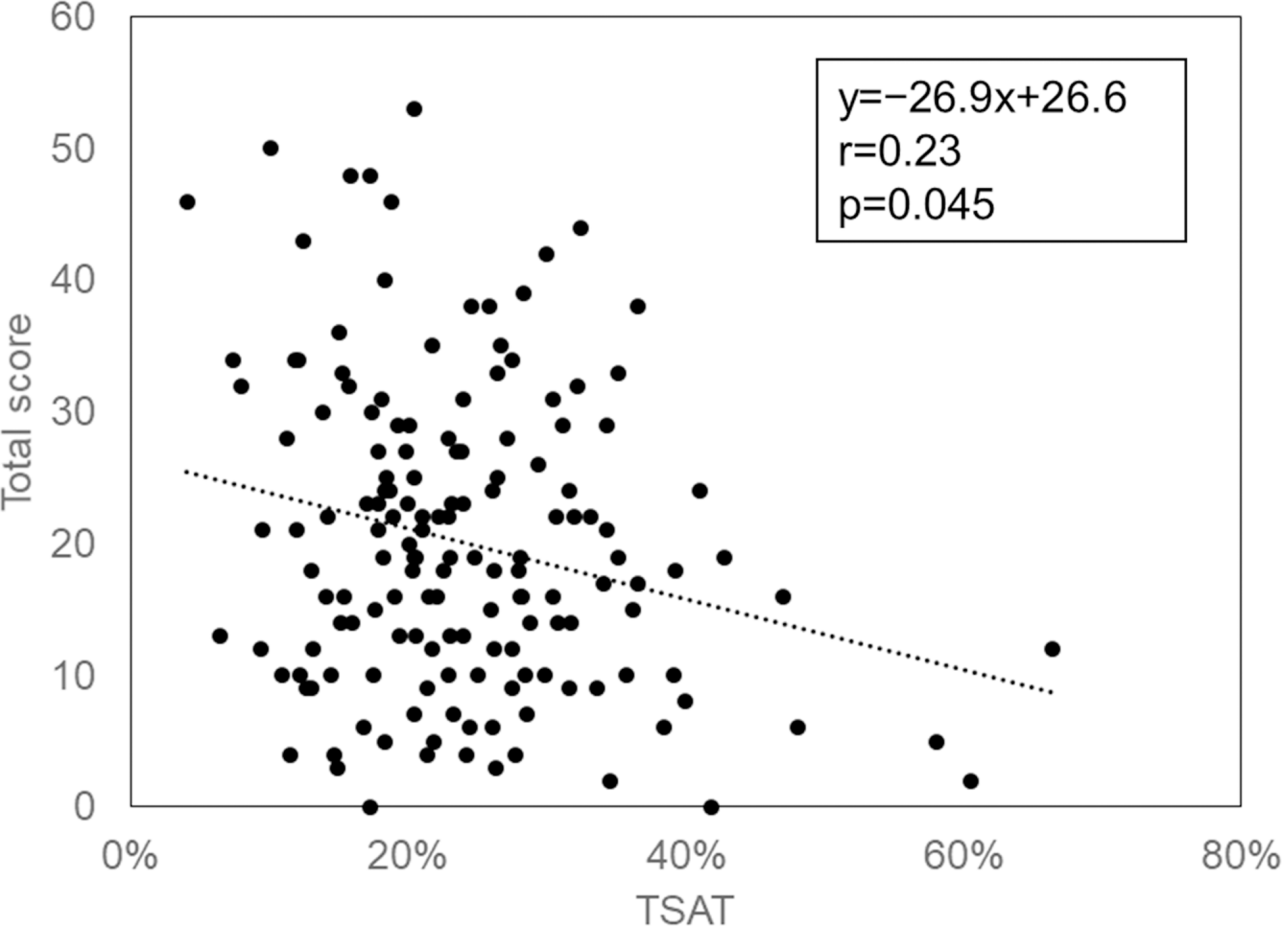
Relationship between TSAT and total score. The strength of linear association between TSAT and total symptom questionnaire score was analyzed by Spearman’s correlation coefficient. TSAT: transferrin saturation.
