# Supplementary material for: Iron deficiency associates with deterioration in several symptoms independently from hemoglobin level among chronic hemodialysis patients
Source: PLoS One. 2018 Aug 2;13(8):e0201662. doi: 10.1371/journal.pone.0201662 (PMC6072073; doi:10.1371/journal.pone.0201662)
Supplement: S1 Fig — (PDF) [file pone.0201662.s001.pdf]

透析治療に関する自覚症状調査シート 3.4

名前：　　　　　　　　　　　　　記入日：平成　　　年　　　月　　　日

該当する顔マークの下の数字に〇をつけてください。

普段の気になる様子について教えてください

1. 関節痛はありますか？

一番痛い部位に〇をつけてください→ 肩・腰・ひざ・その他（　　　　　　　）

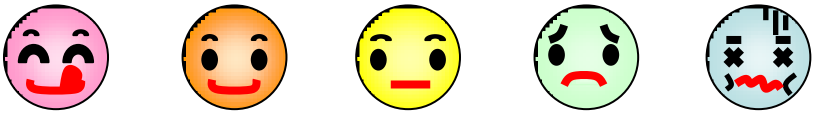

0　1　2　3　4

まったくない ⟷ 強くある

2. からだのかゆみはありますか？（シャント周辺は除く）

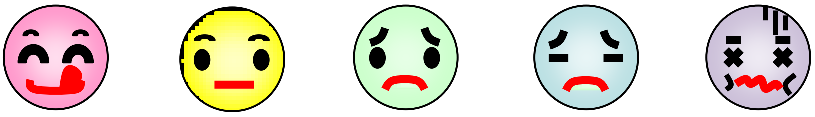

0	1	2	3	4
まったくない	いくらか	かなり	相当	ひどい

3. イライラを感じることはありますか？

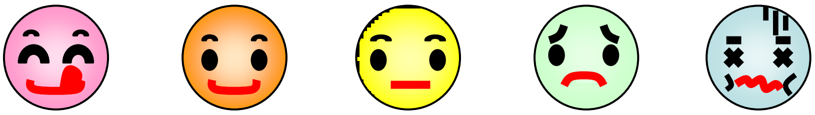

0　1　2　3　4

まったくない ⟷ 強く感じる

4. だるさを感じますか？

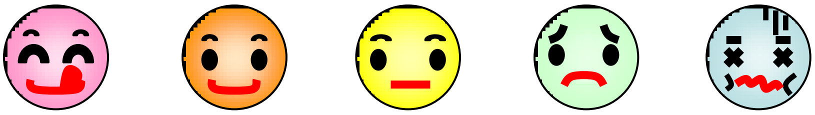

0　1　2　3　4

まったくない ⟷ 強く感じる

5.　動悸や息切れがありますか？

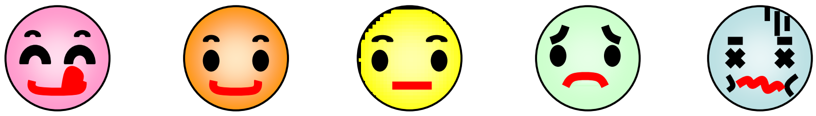

0　1　2　3　4

まったくない ⟷ ひんぱんにある

6.　便秘でお悩みですか？

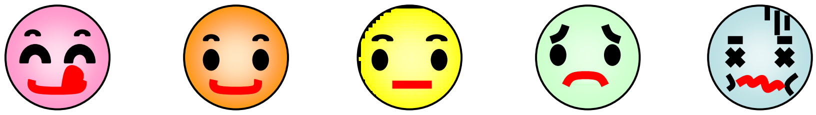

0　1　2　3　4

悩んでいない ⟷ とても悩んでいる

●下剤を飲んでいますか？（✓をつけてください）

□ 飲まない　　□ ときどき飲む　　□ 毎日飲む

7.　ふとんに入ってすぐに寝つけますか？

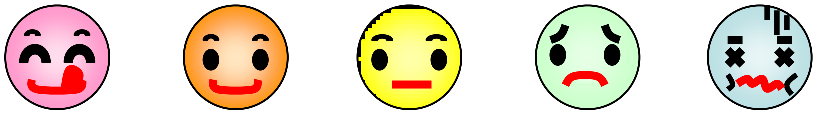

0　1　2　3　4

すぐに寝つける ⟷ なかなか寝つけない

8.　朝までぐっすり眠れますか？

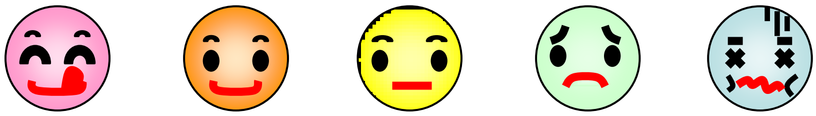

0　1　2　3　4

眠れる ⟷ ほとんど眠れない

●睡眠薬を飲んでいますか？（✓をつけてください）

□ 飲まない　　□ ときどき飲む　　□ 毎日飲む

Pod
Patient oriented dialysis

透析について教えてください

9. 穿刺痛はありますか？

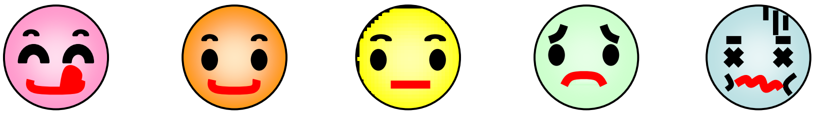

0 まったくない / 1 いくらか / 2 がまんできるくらい / 3 かなり / 4 がまんできないほど痛い

10. 透析中、透析前後で頭痛はありますか？

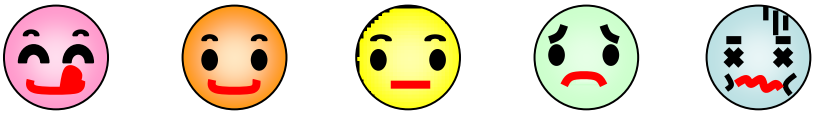

0 1 2 3 4

まったくない ←→ 毎回ある

11. 透析中に苦痛をともなう血圧低下がありますか？

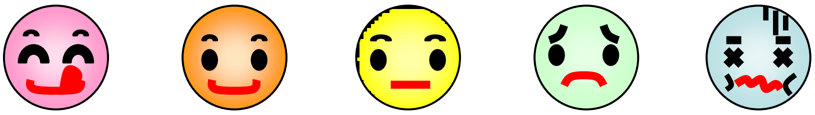

0 1 2 3 4

まったくない ←→ 毎回ある

12. 透析中に足のつり（こむらがえり）はありますか？

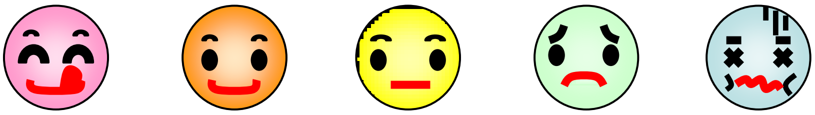

0 1 2 3 4

まったくない ←→ 毎回ある

13. 透析終了後すぐにベッドから起き上がることができますか？

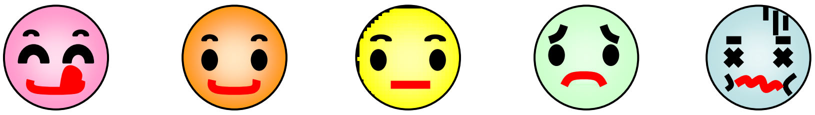

0 1 2 3 4

すぐ起き上がれる ←→ かなりの時間起き上がれない

Pod
Patient oriented dialysis

食生活について教えてください

14. 食欲はありますか？

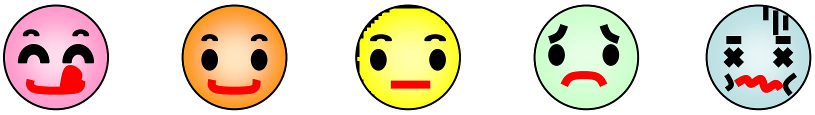

0　1　2　3　4

とてもある ⟷ まったくない

15. 食事はおいしいですか？

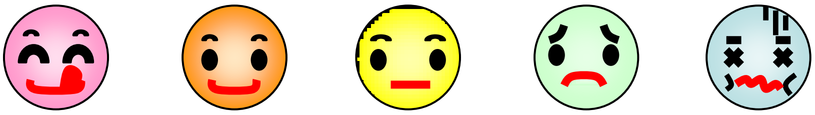

0　1　2　3　4

とてもおいしい ⟷ まったくおいしくない

16. のどは渇きますか？

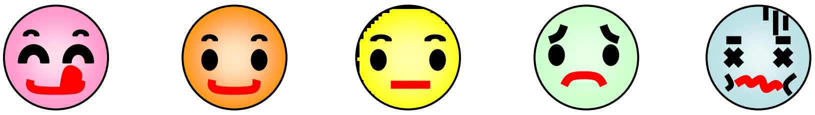

0　1　2　3　4

まったく渇かない ⟷ とても渇く

17. 食事制限はつらいですか？

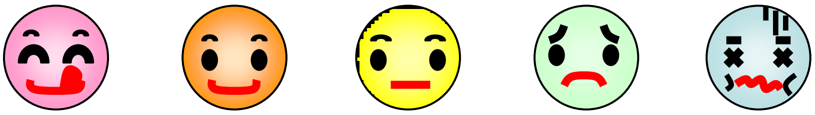

0　1　2　3　4

まったくつらくない ⟷ とてもつらい

日々の気分を教えてください

18. 最近、ゆううつな気分または沈んだ気持ちになりますか？
（✓をつけてください）

☐ ならない　　☐ なる

19. 最近、何事も興味がわかない、いつも楽しめていたことが楽しめないことがありますか？（✓をつけてください）

☐ ない　　☐ ある

さいごに

20. 今の自分の生活に満足していますか？

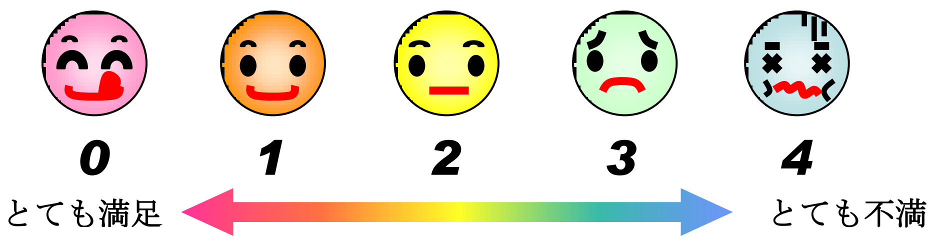

その他、気になることや質問がございましたら、お書きください。

評価シートへのご記入、お疲れ様でした。

何か心配なことがありましたら、いつでもスタッフにご相談ください。
